# Clinical outcomes in pediatric acute lymphoblastic leukemia: a retrospective cohort study from a tertiary center in Indonesia

**DOI:** 10.3389/fped.2026.1830355

**Published:** 2026-09-16

**Authors:** Dian Puspita Sari, Dewi Rosariah Ayu, R. M. Indra, Eny Rahmawati

**Affiliations:** 1 Department of Oncology, Dr. Mohammad Hoesin General Hospital, Palembang, Indonesia; 2 Department of Mother and Child, Dr. Mohammad Hoesin General Hospital, Palembang, Indonesia; 3 Department of Clinical Pathology, Dr. Mohammad Hoesin General Hospital, Palembang, Indonesia

**Keywords:** acute lymphoblastic leukemia, children, immunophenotypes, outcomes, prognostic factors

## Abstract

**Introduction:**

Acute lymphoblastic leukemia (ALL) is the most common pediatric malignancy, accounting for approximately 80% of childhood acute leukemia cases. Despite substantial improvements in survival in high-income countries, outcomes in Indonesia remain suboptimal.

**Methods:**

This retrospective cohort study analyzed medical records of pediatric ALL patients treated at Dr. Mohammad Hoesin General Hospital, Palembang, from 2019 to 2023 to describe demographic, clinical, laboratory, and immunophenotypic characteristics and their association with overall survival.

**Results:**

Most patients were aged 1–10 years, male, and had normal nutritional status, with leukocyte counts <50,000/µL, thrombocytopenia, and no central nervous system involvement or mediastinal mass. B-lineage ALL predominated. Univariate survival analysis showed that age at diagnosis, nutritional status, mediastinal mass, leukocyte count, and risk stratification were significantly associated with overall survival, whereas immunophenotypic classification was not. Multivariate Cox regression identified age >10 years and poor nutritional status as independent predictors of poorer overall survival.

**Conclusion:**

These findings underscore the importance of early diagnosis and nutritional optimization to improve overall survival in pediatric ALL.

## 1 Introduction

Acute leukemia in children comprises Acute Lymphoblastic Leukemia (ALL) and Acute Myeloid Leukemia (AML), with ALL accounting for approximately 80% of pediatric acute leukemia cases and occurring about five times more frequently than AML (1). Acute Lymphoblastic Leukemia (ALL) is the most common childhood malignancy, with survival rates having improved substantially over the past two decades. However, in Indonesia, survival remains relatively low, ranging from 20% to 60%. Early deaths most frequently occur during or prior to the induction phase, underscoring the importance of prognostic factors such as initial leukocyte count, mediastinal or central nervous system involvement, and response to induction therapy in risk stratification (2). Genetic heterogeneity in ALL has led to the identification of new subtypes that improve risk stratification but also increase the complexity of diagnosis and therapeutic decision-making. Consequently, accurate diagnostic procedures, including morphological analysis, immunophenotypes, and cytogenetic evaluation, are crucial for optimal treatment planning (3).

Five-year survival rates in children with ALL show marked disparities between high-income countries and low- to middle-income countries. Over recent decades, overall survival for pediatric ALL has improved significantly in developed countries. Data from the Surveillance, Epidemiology, and End Results (SEER) program indicate that the five-year survival rate for patients aged 0–19 years reached approximately 86.3% during the 2015– 2021 period (4). In Canada, survival rates are even higher, reaching 94% in pediatric populations (5). In the United States and most European countries, five-year survival rates range from 85% to 90%, whereas in Southeast Asia, the highest survival rates have been reported in Malaysia (69.4%) and Thailand (55.1%). In contrast, rates in Indonesia remain lower, with studies from Dharmais Cancer Hospital and Dr. Sardjito General Hospital Yogyakarta reporting five-year survival rates of 28.9% and 31.8%, respectively (6).

Immunophenotyping is a method used to identify the phenotypic profile of a cell population through specific antigen–antibody interactions. Flow cytometry is the primary approach for immunophenotyping in hematologic malignancies, serving not only to establish diagnosis but also to monitor minimal residual disease and assess therapeutic response during and after treatment (7).

Acute leukemia was initially classified into four major groups based on immunophenotypes and later evolved into multiple subgroups. In ALL, leukemic cells undergo rearrangements of immunoglobulin and T-cell receptor genes, allowing classification into B-lineage ALL (B-ALL) and T-lineage ALL (T-ALL). B-ALL is typically characterized by expression of CD19, CD79a, and CD22, with variable expression of CD34 or HLA-DR, and CD10, whereas T-ALL is usually defined by cytoplasmic CD3 with variable expression of CD2, CD5, and CD7. Certain immunophenotypic subtypes of ALL are recognized as having higher-risk biological behavior and less favorable clinical outcomes. In B-lineage ALL, pro-B ALL is frequently associated with KMT2A rearrangements and poorer prognosis compared with common B-ALL. Likewise, immature T-cell subtypes, particularly early T-cell precursor (ETP) ALL, have been associated with induction failure, higher relapse rates, and inferior overall survival, reflecting the biological heterogeneity of pediatric ALL (8–11).

Previous studies from high-income countries have demonstrated substantial improvements in pediatric ALL outcomes, with five-year survival rates exceeding 85%–90% due to advances in molecular diagnostics, risk-adapted therapy, and supportive care. In contrast, studies from low- and middle-income countries (LMICs) continue to report poorer outcomes, often associated with delayed diagnosis, treatment-related toxicity, malnutrition, infection, and treatment abandonment. Several studies in Indonesia have primarily focused on incidence and mortality patterns, while data integrating immunophenotypic characteristics with clinical outcomes and survival in real-world settings remain limited. Therefore, further studies from resource-limited settings are needed to better understand the interplay between biological and non-biological prognostic factors in pediatric ALL (12–14).

Despite the central role of immunophenotypes in the diagnostic workup of pediatric acute lymphoblastic leukemia (ALL), evidence regarding its association with clinical outcomes in real-world settings with high early mortality remains limited, particularly in low- and middle-income countries. In such settings, non-biological factors such as nutritional status, delayed diagnosis, treatment abandonment, and limited supportive care may exert a stronger influence on outcomes than immunophenotypic classification alone. Furthermore, data from Indonesia remain scarce, especially studies integrating immunophenotypic profiles with demographic, clinical, and laboratory characteristics in relation to overall survival. Therefore, this study aimed to describe the demographic, clinical, laboratory, and immunophenotypic characteristics of pediatric ALL patients and to evaluate the prognostic relevance of immunophenotypic and clinical factors in relation to overall survival in a tertiary referral center in Indonesia.

## 2 Material & methods

This retrospective cohort study evaluated demographic, clinical, laboratory, and immunophenotypic factors associated with overall survival among pediatric patients with acute lymphoblastic leukemia (ALL). The study was conducted at the Department of Pediatrics, Faculty of Medicine, Universitas Sriwijaya, Dr. Mohammad Hoesin General Hospital Palembang, from February to August 2025, using medical records from 2019 to 2023.

The study population included pediatric ALL patients aged 1–18 years who received chemotherapy according to the 2018 ALL treatment protocol. Patients with incomplete medical records were excluded from the study. Patients who discontinued treatment or were lost to follow-up were retained for survival analysis. Patients were considered lost to follow-up if they discontinued chemotherapy without subsequent documented clinical follow-up or if no further clinical information was available for at least 6 months after their last documented chemotherapy. Those without documented death were treated as censored observations according to their last documented follow-up. Follow-up duration was calculated from the date of ALL diagnosis to the date of death, last documented clinical follow-up, or the end of the study period (31 December 2023), whichever occurred first. Overall survival (OS) was defined as the time from diagnosis to death from any cause. Patients who remained alive at the end of follow-up were censored at their last documented follow-up date.

Risk stratification was classified according to the Indonesian Childhood ALL-2018 Protocol into standard-risk and high-risk groups. According to protocol-defined criteria, patients were categorized as high-risk if they had one or more of the following characteristics: age <1 year or >10 years, initial leukocyte count ≥50,000/µL, mediastinal mass, central nervous system involvement, T-cell immunophenotyping or poor response to induction therapy. Patients who did not meet these criteria were categorized as standard-risk.

Variables analyzed included age at diagnosis, sex, leukocyte count, hemoglobin level, platelet count, immunophenotypic subtype, risk stratification, central nervous system involvement, mediastinal mass, nutritional status, and overall survival. Immunophenotypic analysis was performed using flow cytometry on bone marrow and/or peripheral blood samples obtained at diagnosis. B-lineage ALL was identified based on expression of markers such as CD19, CD10, CD20, CD22, CD34, and HLA-DR, whereas T-lineage ALL was characterized by expression of cytoplasmic CD3, CD2, CD5, and CD7. Antigen expression was considered positive when ≥20% of leukemic cells expressed the respective marker. Immunophenotypic classification was established according to institutional laboratory standards and interpreted by clinical pathologists experienced in hematologic malignancies.

All patients received chemotherapy according to the Indonesian Childhood ALL-2018 Protocol, consisting of induction, consolidation, reinduction, and maintenance phases. Supportive therapy, including blood transfusion, antimicrobial treatment, and nutritional management, was administered according to institutional standards and individual clinical conditions.

Molecular diagnostic testing, including cytogenetic analysis, fluorescence *in situ* hybridization (FISH), and fusion transcript analysis by reverse transcription polymerase chain reaction (RT-PCR), was not available. This limitation reflects the challenges commonly encountered in resource-limited settings.

Data were analyzed using IBM SPSS Statistics version 26 (IBM Corp., Armonk, NY, USA). Descriptive statistics were used to summarize demographic, clinical, laboratory, and immunophenotypic characteristics of the study population. Categorical variables were presented as frequencies and percentages, while continuous variables were summarized using means and standard deviations or medians and interquartile ranges, as appropriate. Survival probabilities were estimated using the Kaplan–Meier method and compared between groups using the log-rank test. Variables with a *p*-value <0.25 in univariate log-rank analysis were included in multivariate Cox proportional hazards regression analysis to identify independent predictors of overall survival. Hazard ratios (HRs) with 95% confidence intervals (CIs) were calculated, and statistical significance was defined as *p* < 0.05. Ethical approval was obtained from the Ethics Committee of Dr. Mohammad Hoesin General Hospital, and all data were anonymized to ensure patient confidentiality.

## 3 Result

This is the flow chart of subject selection of this research and association between Demographic Characteristics and Overall Survival in Pediatric ALL (Figure 1).

**Figure 1:**
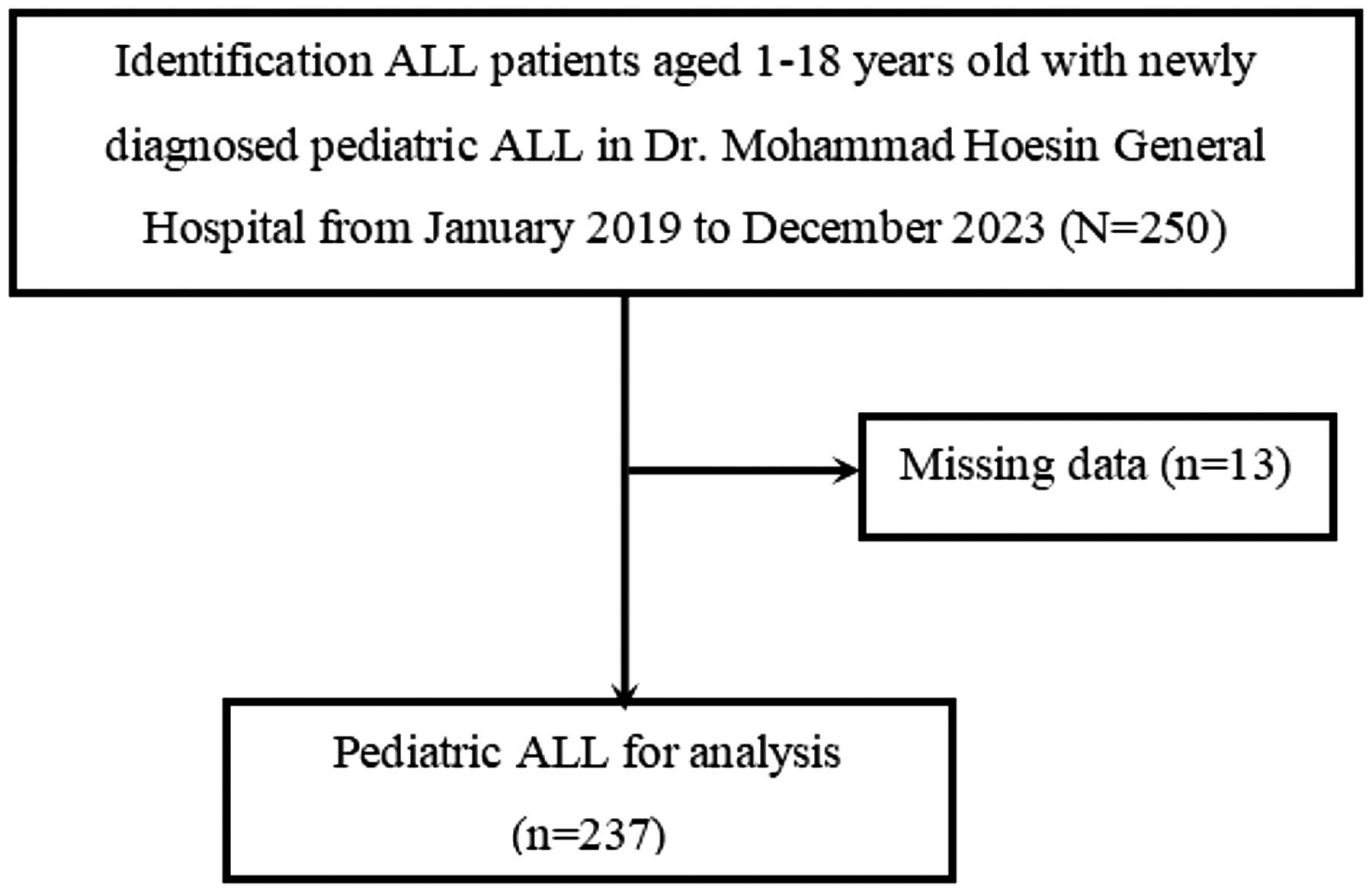
Flow chart of subject selection.

A total of 237 pediatric patients with acute lymphoblastic leukemia (ALL) were included in the analysis. The baseline clinical and immunophenotypic characteristics of the patients are presented in Table 1. The distribution of demographic characteristics, clinical findings at diagnosis, laboratory parameters, immunophenotypic findings, and risk stratification is summarized in the table.

**Table 1:** Baseline clinical and immunophenotypic characteristics of patients with acute lymphoblastic leukemia.

Variables	Category	*n* (%)
Age	1–10 years	165 (69.6)
	>10 years	72 (30.4)
Gender	Male	140 (59.1)
	Female	97 (40.9)
Nutritional status	Normal	149 (62.9)
	Moderate	67 (28.3)
	Severe	8 (3.4)
	Overweight	13 (5.5)
CNS involvement	Negative	225 (94.9)
	Positive	12 (5.1)
Mediastinal mass	No	233 (98.3)
	Yes	4 (1.7)
Leukocyte count	<50,000	181 (76.4)
	≥50,000	56 (23.6)
Hemoglobin	≥6	173 (73.0)
	<6	64 (27.0)
Platelet count	≥50,000	52 (21.9)
	<50,000	185 (78.1)
Immunophenotype	B-lineage	213 (89.9)
	T-lineage	24 (10.1)
Risk stratification	Standard-risk	102 (43.0)
	High-risk	134 (57.0)

Table 2 shows the univariate survival analysis of demographic, clinical, laboratory, and immunophenotypic characteristics in pediatric ALL. Age at diagnosis >10 years, poor nutritional status, presence of mediastinal mass, elevated leukocyte count, and high-risk stratification were significantly associated with poorer overall survival (log-rank *p* = 0.001, 0.010, 0.031, 0.027, and 0.004, respectively). No significant differences in overall survival were observed according to gender, CNS involvement, hemoglobin level, platelet count, or immunophenotypic lineage.

**Table 2:** Univariate analysis of factors associated with overall survival in pediatric acute lymphoblastic leukemia.

Variables	Category	Mean Survival (months)	*p*-value
Age	1–10 years	31.018	0.001
	>10 years	15.912	
Gender	Male	28.915	0.244
	Female	23.890	
Nutritional status	Normal	30.150	0.010
	Moderate	26.739	
	Severe	12.038	
	Overweight	10.869	
CNS involvement	Negative	28.008	0.368
	Positive	15.849	
Mediastinal mass	No	28.242	0.031
	Yes	8.713	
Leukocyte count	<50,000	29.612	0.027
	≥50,000	20.060	
Hemoglobin	≥6	28.298	0.467
	<6	21.626	
Platelet count	≥50,000	22.548	0.758
	<50,000	28.452	
Immunophenotype	B-lineage	28.436	0.226
	T-lineage	16.242	
Risk stratification	Standard-risk	33.034	0.004
	High-risk	22.363	

Analysis using log-rank test.

Table 3 presents the multivariate Cox proportional hazards regression analysis. Age at diagnosis and nutritional status were identified as independent predictors of poorer overall survival. Older age was associated with a significantly higher hazard of death (HR 1.707; 95% CI 1.149–2.536; *p* = 0.008), while poorer nutritional status was independently associated with worse overall survival (HR 1.273; 95% CI 1.036–1.565; *p* = 0.022).

**Table 3:** Multivariate Cox regression analysis of factors associated with overall survival in pediatric acute lymphoblastic leukemia.

Variables	HR	95% CI	*p*-value
Age	1.707	1.149–2.536	0.008
Gender	1.449	0.970–2.166	0.070
Nutritional status	1.273	1.036–1.565	0.022
Mediastinal mass	1.661	0.516–5.348	0.395
Leukocyte	1.526	0.969–2.402	0.068
Immunophenotype	1.060	0.556–2.017	0.860

Analysis using multivariate cox regression.

The median follow-up duration was 8.8 months (IQR, 1.41–21.42 months). Overall, 127 of 237 patients (53.6%) were censored, while 110 patients (46.4%) experienced death during the follow-up period. The proportion of censored observations was higher among patients in the standard-risk group (65/102, 63.7%) than among those in the high-risk group (62/135, 45.9%). Conversely, death occurred in 36.3% of patients in the standard-risk group and 54.1% of those in the high-risk group (Table 4).

**Table 4:** Distribution of censored observations and death events by risk stratification.

Risk Stratification	Censored (%)	Death (%)	Total (%)
Standard Risk	65 (63.7%)	37 (36.3%)	102
High Risk	62 (45.9%)	73 (54.1%)	135
Total	127 (53.6%)	110 (46.4%)	237

To further characterize overall survival over time, overall survival was analyzed using the Kaplan–Meier method. The estimated mean overall survival was 27.74 months (95% CI: 24.29–31.19 months), while the median overall survival was 22.57 months (95% CI: 10.59–34.55 months). The Kaplan–Meier survival curve is presented in Figure 2.

**Figure 2:**
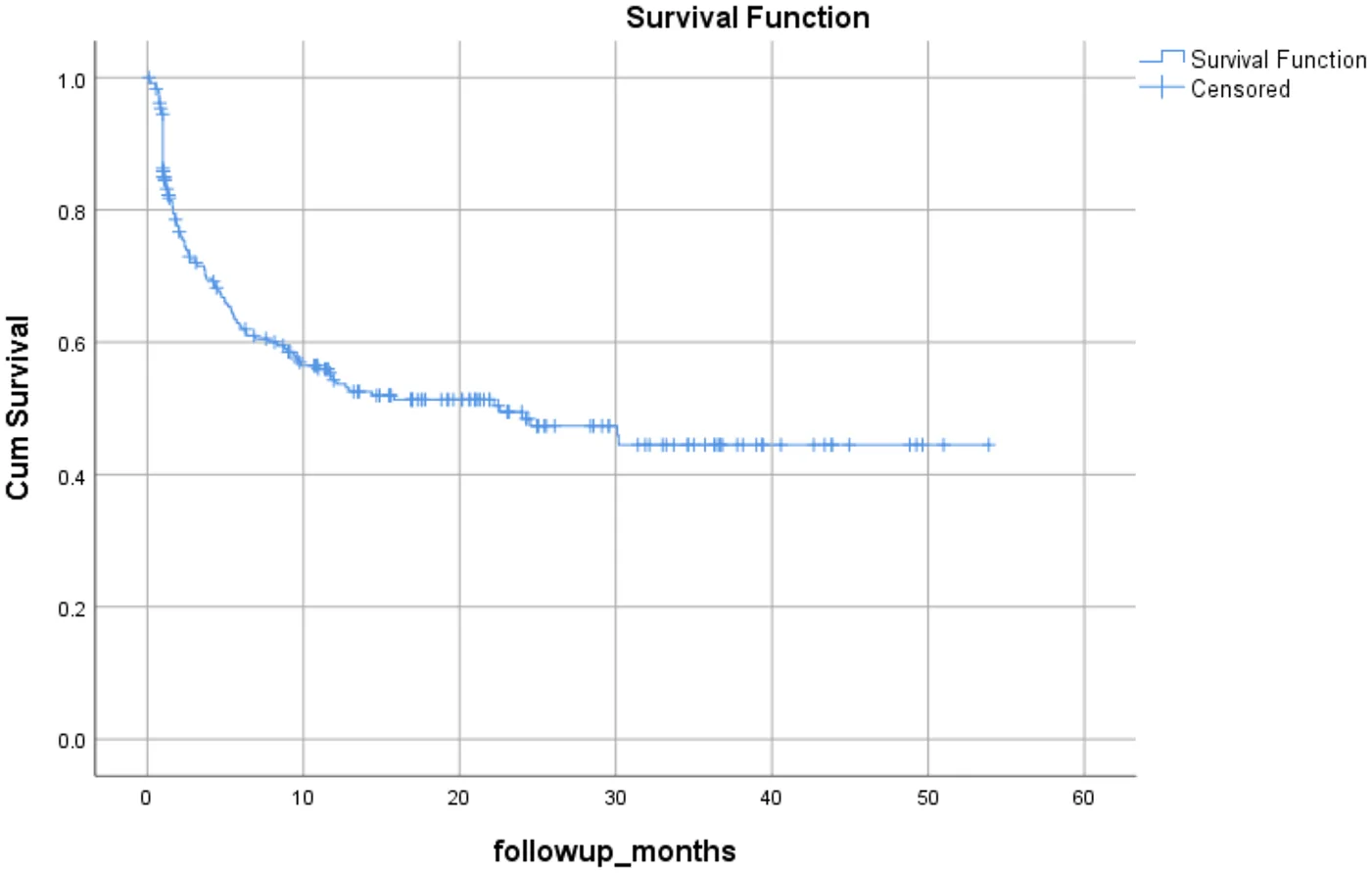
Kaplan–Meier overall survival curve among pediatric patients with acute lymphoblastic leukemia.

Kaplan–Meier survival curves stratified by age and nutritional status are presented in Figures 3, 4. Patients aged >10 years and those with poorer nutritional status showed significantly lower survival probabilities over time.

**Figure 3:**
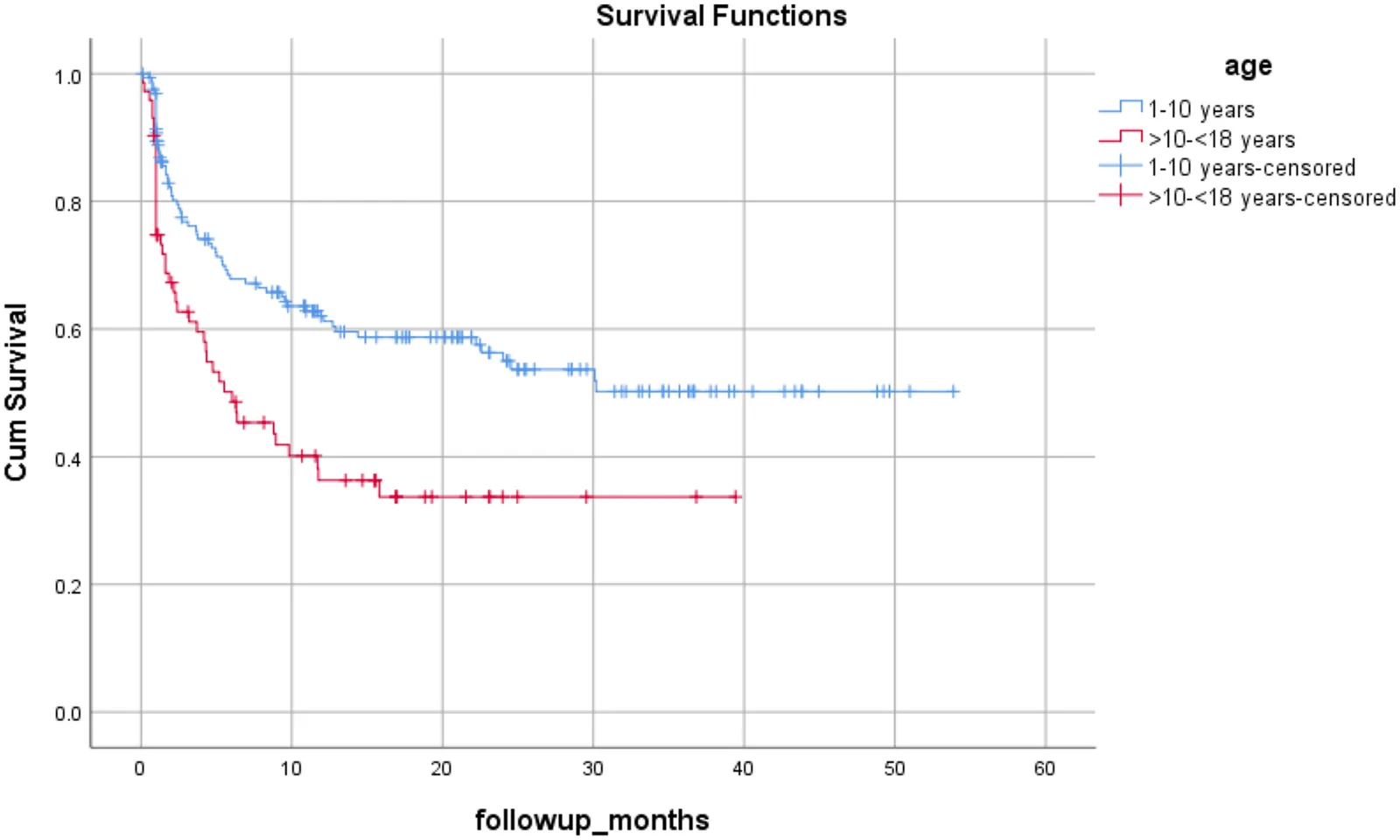
Kaplan–meier survival curves according to age group.

**Figure 4:**
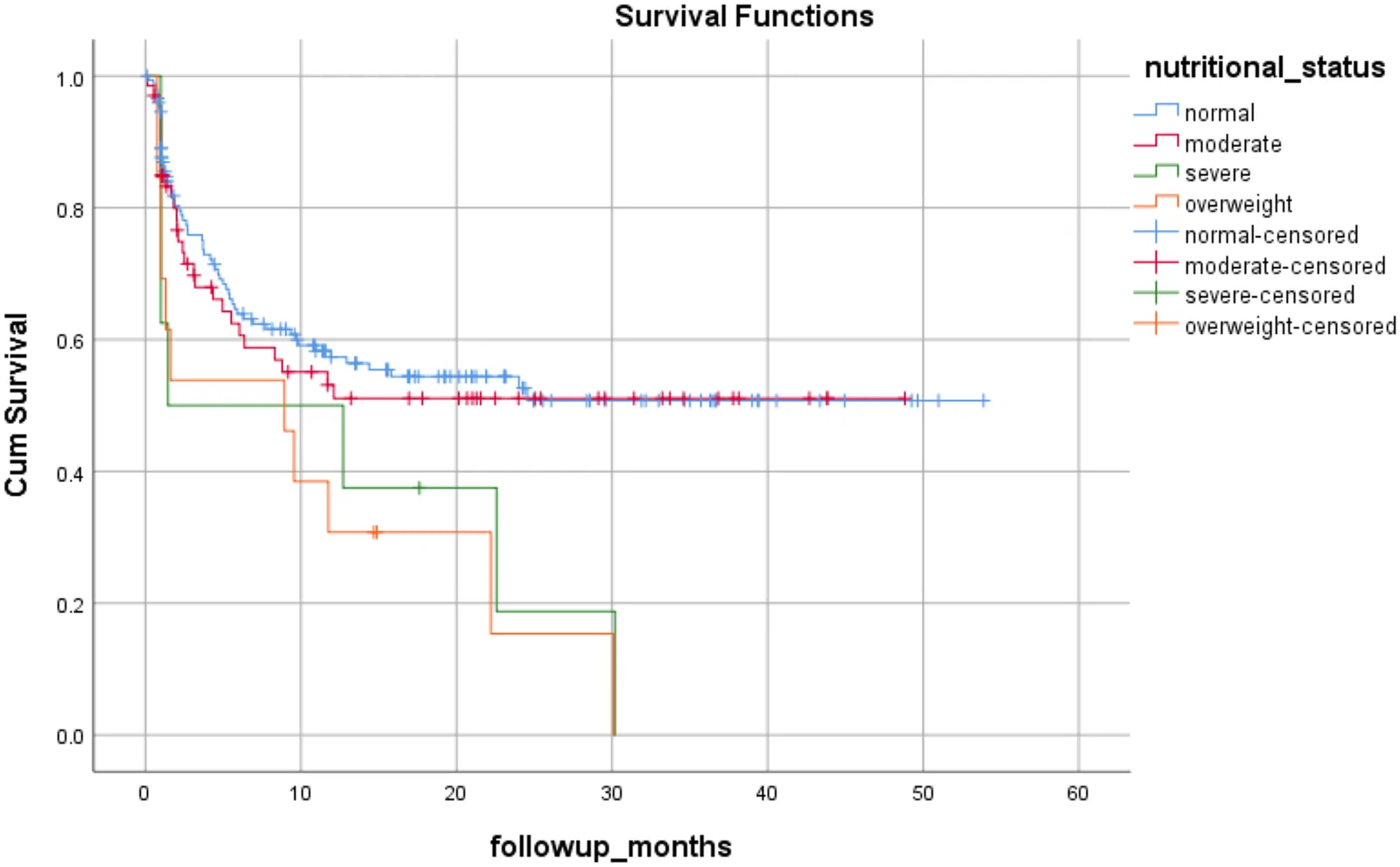
Kaplan–meier survival curves according to nutritional status.

Kaplan–Meier analysis showed no significant difference in overall survival between patients with B-lineage and T-lineage ALL (log-rank *p* = 0.226) (Figure 5).

**Figure 5:**
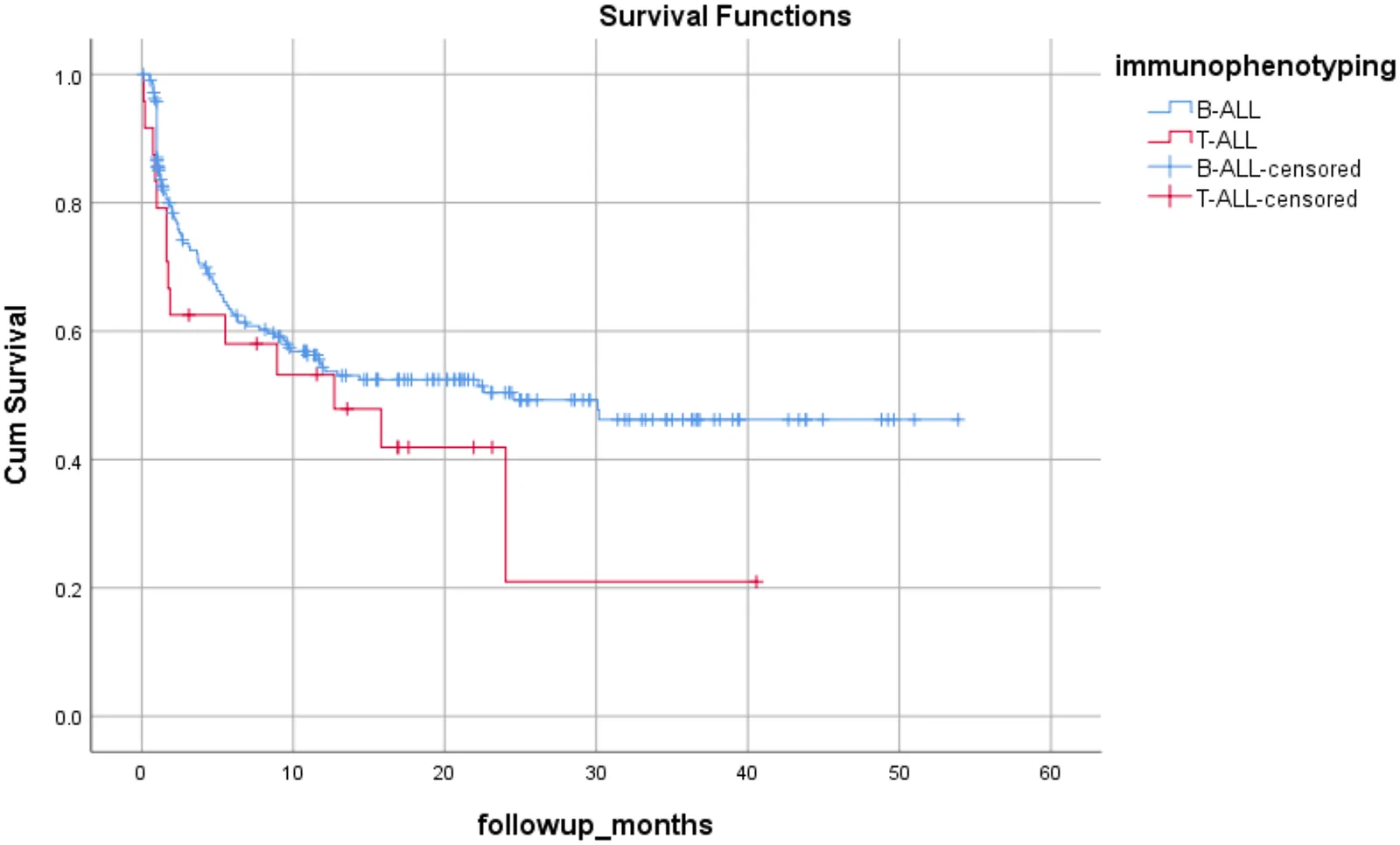
Kaplan–meier survival curves according to immunophenotypic lineage in pediatric acute lymphoblastic leukemia.

## 4 Discussion

This study demonstrated that age at diagnosis and nutritional status were the main independent predictors of overall survival in pediatric acute lymphoblastic leukemia (ALL), whereas immunophenotypic classification was not significantly associated with overall survival. These findings highlight the importance of host-related and clinical factors in determining prognosis, particularly in resource-limited settings. Most pediatric patients with ALL were diagnosed between the ages of 1 and 10 years, consistent with previous reports from Indonesia and other regions (15). The median age at diagnosis (81 months, approximately 6.8 years) aligns with global epidemiological data showing a peak incidence of pediatric ALL during early school age (16). This age distribution reflects a critical period of lymphoid maturation, particularly active proliferation of B-cell precursors, which facilitates the accumulation of postnatal secondary genetic events in children with predisposing lesions such as the ETV6–RUNX1 fusion (17, 18). In addition, the delayed infection hypothesis suggests that postponed immune challenges during early childhood may contribute to leukemogenesis through dysregulated immune responses (19). Importantly, our prognostic analysis demonstrated that age >10 years at diagnosis was associated with poorer overall survival, likely reflecting differences in leukemia biology, treatment responsiveness and tolerance and lymphoblast maturation in older children (4, 20). Older patients are more likely to have unfavorable disease characteristics and may experience greater treatment-related toxicity and resistance, potentially contributing to poorer outcomes (21).

Most patients had normal nutritional status at diagnosis, consistent with previous local studies (22, 23). Nevertheless, severe malnutrition emerged as an independent predictor of poorer overall survival. This finding reinforces evidence that nutritional status plays a crucial role in determining outcomes in pediatric ALL. Malnutrition may exacerbate disease severity by impairing immune competence, reducing skeletal muscle function, and increasing susceptibility to treatment-related toxicity (24). In addition, inadequate nutritional status may compromise host defense against infections through disturbances in cytokine and hormonal regulation and insufficient micronutrient availability. Metabolic alterations associated with malnutrition may also affect drug pharmacokinetics and pharmacodynamics, potentially influencing the efficacy and tolerability of anticancer therapy (25). Furthermore, nutritional status at diagnosis and its evolution during therapy influence disease progression and treatment tolerance and are shaped by cancer-related metabolic alterations, chemotherapy side effects, and socioeconomic factors (26).

At diagnosis, most patients presented with leukocyte counts <50,000/µL, thrombocytopenia, and a substantial proportion had severe anemia, consistent with previous studies (23, 27). These laboratory abnormalities reflect extensive bone marrow infiltration and impaired hematopoiesis, which may affect clinical stability and chemotherapy tolerance (7, 24). Thrombocytopenia, in particular, increases the risk of bleeding complications (28, 29). However, in this study, hemoglobin level and platelet count were not significantly associated with overall survival, suggesting that their prognostic value may be outweighed by other clinical and biological factors.

Central nervous system (CNS) involvement at diagnosis was uncommon in this cohort, in line with prior reports indicating a prevalence of 3%–8% (20, 30). Leukemic CNS infiltration may occur through meningeal invasion, choroid plexus involvement, or hematogenous spread and has been associated with T-lineage phenotype and high leukocyte counts (30). Nonetheless, CNS involvement did not show a significant association with overall survival in the present study.

Mediastinal mass was identified exclusively in patients with T-lineage ALL, consistent with its well-established association with this immunophenotype (31). Extramedullary manifestations such as hepatomegaly, splenomegaly, and lymphadenopathy were common and reflect leukemic infiltration of the reticuloendothelial system (32). Mediastinal mass was significantly associated with poorer overall survival in univariate analysis but lost significance after multivariate adjustment, suggesting that its effect may be influenced by other clinical factors.

Risk stratification remains a cornerstone of ALL management, integrating age, leukocyte count, cytogenetic features, and early treatment response to guide therapeutic intensity (33). In this study, most patients were classified as high risk, and this group exhibited significantly poorer overall survival compared with standard-risk patients, underscoring the prognostic relevance of risk stratification (2, 24).

Nearly half of the patients died during follow-up. The high proportion of early deaths may have substantially influenced the observed overall survival estimates, as deaths occurring during the early phases of treatment contributed disproportionately to the decline in survival probability, particularly during the initial period of follow-up. Most deaths occurred during the early phases of treatment and were primarily related to infectious complications and disease progression, emphasizing the vulnerability of pediatric ALL patients during intensive chemotherapy in resource-limited settings (3, 20). Several factors may contribute to these poorer outcomes in low- and middle-income countries, including delayed diagnosis, limited access to molecular diagnostics, treatment abandonment, infectious complications, malnutrition, and restricted supportive care resources. These challenges may increase early mortality and reduce treatment tolerance compared with high-income settings. Relapse occurred in a minority of patients, predominantly as very early relapse, consistent with previous findings that early relapse remains a major therapeutic challenge (34). Conversely, a proportion of patients achieved remission, highlighting heterogeneity in treatment response influenced by leukemic genetic profiles as well as host-related pharmacodynamic and pharmacogenomic factors (35).

Immunophenotyping revealed a predominance of B-lineage ALL, consistent with global reports indicating that approximately 85% of pediatric ALL cases arise from B-cell lineage, while 10%–15% are T-lineage (36). Although T-lineage ALL is generally associated with older age at presentation and poorer prognosis, immunophenotypic classification was not significantly associated with overall survival in this study (4, 28, 37). This finding differs from several studies in high-income settings where immunophenotypic and molecular subtypes demonstrate stronger prognostic significance, suggesting that, in resource-limited settings, particularly regarding the limitations of supportive treatment, such as prevention and management of infections, the availability of blood products for transfusion, nutritional intervention, and host factors may exert a more dominant influence on overall survival.

Overall, multivariate Cox regression analysis confirmed that age at diagnosis and nutritional status were independently associated with overall survival in pediatric ALL. These findings emphasize that older age at presentation and poor nutritional status remain important determinants of inferior survival, likely through their combined effects on disease biology, treatment tolerance, and susceptibility to treatment-related complications (4, 20, 33).

This study provides real-world evidence from a tertiary referral center in Indonesia regarding the interaction between immunophenotypic, clinical, and host-related factors in pediatric ALL. Unlike studies from high-income countries that primarily emphasize molecular and cytogenetic risk stratification, our findings highlight the substantial influence of non-biological determinants such as nutritional status and treatment-related vulnerability in resource-limited settings. These findings underscore the importance of contextualizing prognostic factors according to healthcare infrastructure and supportive care availability.

Several limitations of this study warrant consideration. The retrospective single-center design reflects real-world clinical practice in a resource-limited setting but may restrict causal inference and generalizability. Molecular diagnostic testing, including cytogenetic and fusion transcript analysis, was not routinely available during the study period, limiting more refined biological risk stratification. Furthermore, detailed immunophenotypic subclassification (e.g., Pro-B, Common-B, Pre-B, or T-cell maturation subtypes) was not routinely documented in the medical records and therefore could not be evaluated. As a result, immunophenotypic analyses were limited to lineage classification and individual antigen expression profiles, which may not fully capture the biological heterogeneity of pediatric ALL. In addition, the high proportion of early deaths and treatment-related complications may have influenced survival estimates and attenuated the observed associations between immunophenotypic characteristics and clinical outcomes. Finally, variations in supportive care access and treatment adherence could not be fully evaluated in this retrospective analysis.

## Data Availability

The raw data supporting the conclusions of this article will be made available by the authors, without undue reservation.
